# Usefulness analysis of the 2018 ASCO/IDSA guideline for outpatient management of fever and neutropenia in adults treated for malignancy

**DOI:** 10.1038/s41598-021-88207-6

**Published:** 2021-09-15

**Authors:** Soyoon Hwang, Ki Tae Kwon, Yoonjung Kim, Sohyun Bae, Hyun-Ha Chang, Shin-Woo Kim, Seung Soo Yoo, Su Youn Nam, Jin Ho Baek

**Affiliations:** 1 Division of Infectious Diseases, Department of Internal Medicine, School of Medicine, Kyungpook National University, Kyungpook National University Hospital, 807, Hoguk-ro, Buk-gu, Daegu, Republic of Korea; 2 Department of Internal Medicine, School of Medicine, Kyungpook National University, Kyungpook National University Hospital, Daegu, Republic of Korea; 3 Gastroenterology, Department of Internal Medicine, Kyungpook National University Hospital, Daegu, Republic of Korea; 4 Department of Oncology/Hematology, Kyungpook National University Chilgok Hospital, School of Medicine, Kyungpook National University, Daegu, Republic of Korea

**Keywords:** Cancer, Infectious diseases

## Abstract

Although the clinical practice guideline for outpatient management of febrile neutropenia (FN) in adults treated for malignancy was updated by the ASCO/IDSA in 2018, most patients with FN in our hospital have been hospitalized. We performed this study to analyze the usefulness of the guideline. The medical records of patients hospitalized for FN in Kyungpook National University Chilgok Hospital from May 2016 to April 2018 were retrospectively reviewed. The feasibility of candidates for outpatient management according to the guideline was evaluated based on the outcomes. A total of 114 patients were enrolled and categorized into two groups, low-risk (38.6%) and high-risk (61.4%). The proportion of feasible candidates for outpatient management was 70.2% and was higher in the low-risk than in the high-risk group (90.0% vs. 57.1%; *P* < 0.001). The low-risk group had no mortality, no resistance to oral amoxicillin/clavulanate or ciprofloxacin, a higher rate of successful empirical antibiotics, and lower rates of glycopeptide or carbapenem administration. A significant number of hospitalized cancer patients treated for FN after chemotherapy were found to be feasible candidates for outpatient management. The guideline can be a useful tool to reduce labor of healthcare workers and hospitalization costs.

## Introduction

In 2018, the American Society of Clinical Oncology (ASCO) and the Infectious Diseases Society of America (IDSA) updated the clinical practice guideline for outpatient management of febrile neutropenia (FN)^1^. However, despite this guideline, most patients with FN in our cancer center have been hospitalized. To our knowledge, the same is true in many Korean hospitals. Research on five major solid tumors (gastric, colon, lung, breast, and ovarian cancers) using the Korean National Health Insurance Corporation claim database from 2003 to 2013 revealed that approximately 70,000 hospitalizations owing to cancer-related FN accounted for 8.5% of all cancer-related hospitalizations, and the average cost per hospitalization for FN was 3,818061 Korean won^2^. In a similar study published in the United States in 2012 using a national inpatient sample database, there were 91,560 hospitalizations for cancer-related FN, accounting for 5.2% of all cancer-related hospitalizations, and the average cost of hospitalizations for cancer-related FN was $24,770 (United States dollars [USD]) per hospitalization^3^. Although medical systems and insurance coverage criteria vary by country, the financial burden of FN care is a problem in many countries as the number of cancer patients increases worldwide^4–6^.

The development of medical technology and improved access to emergency medical services have created an overcrowding problem in emergency rooms (ER)^7,8^. In Korea, according to the Ministry of Health and Welfare’s “2015 Emergency Medical Agency Assessment,” the average length of stay in the ER at the nation’s 20 leading hospitals was 14 h, which is more than three times longer than the 4-h target for ER stays suggested by the United Kingdom’s National Health Service^9,10^. FN patients are often hospitalized through the ER, and overcrowding in the ER can cause discomfort and increase the risk of patient safety incidents^3,11^. In addition, hospitalization periods in Korea are longer than in other countries^12^, and the number of doctors and nurses caring for inpatients is also insufficient^13,14^. Considering patient safety, overcrowded ERs, and the burden on healthcare workers and finances, patients who are suitable for outpatient management should be treated on an outpatient basis rather than hospitalized. However, it may be difficult for the 2018 ASCO/IDSA guideline to be applied directly in our cancer center because of different circumstances in each country and region. Few studies have been conducted on the practical application of this guideline in clinical practice for patients with FN. We conducted this study to determine the proportion of hospitalized patients who could be treated in an outpatient setting if the 2018 ASCO/IDSA guideline were implemented. We analyzed the feasibility of candidates for outpatient management according to the guideline by assessing the treatment outcomes of the patients.

## Methods

### Study design

We conducted a retrospective cohort study at a tertiary-care academic cancer center. We reviewed the electronic medical records to analyze all hospitalized patients with FN between May 1, 2016 and April 1, 2018. Eligible patients included those who were at least 18 years old with a diagnosis of cancer who received chemotherapy, regardless of the cancer stage or chemotherapy regimens. If the same patient were hospitalized more than once during the study period, only the first admission was included. The Institutional Review Board of Kyungpook National University Chilgok Hospital (IRB File no. 2019-07-003) approved all experimental protocols of this study and waived the requirement to obtain any informed consent in July 2019. All methods of this study were carried out in accordance with the 1964 Declaration of Helsinki.

Information regarding the clinical and demographic characteristics of the patients, including age, sex, type of malignancy, hospitalization route, history of intensive care unit stay, duration of neutropenia after chemotherapy, laboratory tests, vital signs, causes of fever, administered antibiotics, duration of fever, duration of neutropenia, duration of hospitalization, and clinical outcome, was collected. In addition, to assess the Multinational Association for Supportive Care in Cancer (MASCC) score, Clinical Index of Stable Febrile Neutropenia (CISNE) score, and Talcott classification, we reviewed the patients’ medical history including comorbidities, Eastern Cooperative Oncology Group performance status, experience of side effects of chemotherapy, and symptoms and presence of dehydration at the time of the visit because of FN. The presence of dehydration was defined when urine specific gravity was 1.020 or greater ^15^. The MASCC score, CISNE score, and Talcott classification of the patients were used to classify the patients into a low-risk group (LRG) and high-risk group (HRG) for medical complications from FN. To analyze the clinical characteristics of candidates for outpatient management according to the ASCO/IDSA guideline, we compared the demographic characteristics, risk scores, clinical features, and outcomes between the LRG and HRG. To evaluate the feasibility of the candidates for outpatient management according to the ASCO/IDSA guideline, the outcome of the administered antibiotic regimen and the feasibility of switching to an oral antibiotic regimen were assessed.

### Definitions

FN was defined as a documented fever of 38.0 °C (100.4°F) or higher and an absolute neutrophil count (ANC) less than 500 cells/mm^3^ or a decrease in ANC to less than 500 cells/mm^3^ during the 24 h after fever onset. According to the 2010 IDSA guideline^16^, fever was defined as a single oral temperature measurement of 38.3 °C (101°F) or higher or a temperature of 38.0 °C (100.4°F) or higher sustained over a 1-h period in FN. However, we prefer to measure the tympanic or axillary temperature rather than oral temperature. Because the duration of sustained fever may be shortened by the use of antipyretic agents, for this study, we defined an episode of FN as ANC less than 500 cells/mm^3^ and a tympanic or axillary temperature of 38.0 °C (100.4°F) or higher at least once.

**Figure 1:**
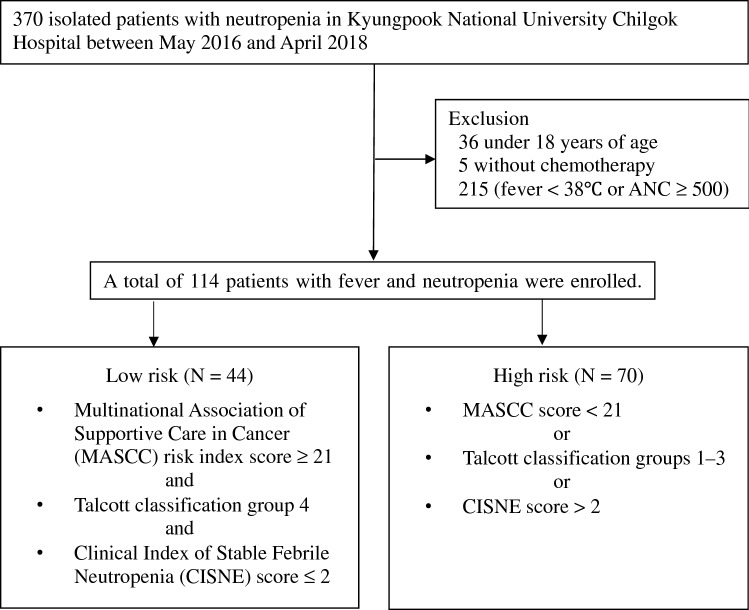
Enrollment and risk classification.

The LRG was defined as having a MASCC score of 21 or higher, Talcott’s classification group IV, and a CISNE score of 0 to 2. The HRG was defined as having a MASCC score of less than 21, Talcott’s classification group I to III, or a CISNE score of ≥ 3 (Fig. 1)^1^. Successful initial empirical antibiotic use was defined as successful recovery from FN without modification of the initial empirical antibiotics. Feasible outpatient management was defined as (1) successful use of initial empirical antibiotics, (2) no carbapenem or glycopeptide administration, and (3) no isolated microorganism resistant to amoxicillin/clavulanate or ciprofloxacin.

### Statistical analysis

To compare various characteristics between the HRG and LRG, categorical variables were analyzed by the chi-square test or Fisher’s exact test and continuous variables were analyzed by Student’s *t* test or Welch’s *t* test. All analyses were performed using the statistical software R (version 3.2.2; R Foundation for Statistical Computing)^17^. Two-sided *P* values less than 0.05 were considered statistically significant.

## Results

### Results among all patients

A total of 114 patients were enrolled, with 44 patients (38.6%) classified as LRG and 70 patients (61.4%) classified as HRG (Fig. 1). The mean age of the study population was 60.8 years (standard deviation [SD], 13 years), and the male to female ratio was 54 to 60. The most common malignancy was lung cancer (25.4%), followed by breast cancer (20.2%), genital cancer (16.7%), lymphoma (13.2%), gastrointestinal malignancy (11.4%), and others. Of the patients, 35.1% were admitted as outpatients and 64.9% visited the ER or were already hospitalized. A total of 13 patients experienced microbiologically defined infection: there were seven cases of *Escherichia coli* (isolated from the blood in six patients and from the urine in one patient), two cases of *Pseudomonas aeruginosa* (isolated from the sputum in one patient and from the urine in one patient), two cases of *Klebsiella pneumonia* (isolated from the blood in both), one case of *Enterobacter aerogenes* (isolated from the urine), and one case of influenza. The most frequently prescribed initial empirical antibiotic regimen was piperacillin/tazobactam (TZP) monotherapy (55.3%). Among all patients, feasibility for outpatient management, successful initial empirical antibiotic use, and mortality were 70.2%, 77.2%, and 11.4%, respectively.

### Comparisons between the LRG and HRG

Tables 1 and 2 show the comparisons of demographic characteristics, laboratory findings, risk scores, clinical features, and outcomes between the LRG and HRG. The patients in the LRG were younger (mean age, 53.1 years; SD 11.8 years) compared with the HRG (mean age, 65.6 years; SD, 11.3 years) (*P* < 0.001). There were more women than men in the LRG (86.4% vs. 31.4%; *P* < 0.001), and they had breast cancer (45.5% vs. 4.3%; *P* < 0.001) and genital cancer (29.5% vs. 8.6%; *P* = 0.008) more frequently than in the HRG. The HRG patients had lung cancer more frequently than the LRG patients (38.6% vs. 4.5%; *P* < 0.001). The LRG patients had a higher platelet count (142,400 vs. 102,600/µL; *P* = 0.012) and lower C-reactive protein (3.7 vs. 15.1 mg/dL; *P* < 0.001) than the HRG patients.

**Table 1:** Comparisons of demographic characteristics, laboratory findings, and risk scores between low-risk and high-risk groups.

Variables	Overall (N = 114)	Low-risk (N = 44)	High-risk (N = 70)	*P*
**Age (years), mean (SD)**	60.8 (13)	53.1 (11.8)	65.6 (11.3)	< 0.001
**Gender, N (%)**				< 0.001
Male	54 (47.4)	6 (13.6)	48 (68.6)	
Female	60 (52.6)	38 (86.4)	22 (31.4)	
**Malignant disease, N (%)**				< 0.001
Lung cancer	29 (25.4)	2 (4.5)	27 (38.6)	< 0.001
Breast cancer	23 (20.2)	20 (45.5)	3 (4.3)	< 0.001
Genital cancer	19 (16.7)	13 (29.5)	6 (8.6)	0.008
Lymphoma	15 (13.2)	5 (11.4)	10 (14.3)	0.869
GI cancer	13 (11.4)	3 (6.8)	10 (14.3)	0.358
Others	15 (13.2)	1 (2.3)	14 (20.0)	0.015
**Laboratory results on admission, mean (SD)**				
WBC count (/µL)	773.2 (484.9)	901.6 (481.0)	692.6 (472.9)	0.024
ANC count (/µL)	172.3 (128.3)	165.0 (112.3)	176.9 (138.0)	0.633
Hemoglobin (g/dL)	10.9 (13.0)	10.1 (1.5)	11.4 (16.6)	0.513
Platelet count (/µL)	117.9 (83.0)	142.4 (81.5)	102.6 (80.7)	0.012
CRP (mg/dL)	10.7 (10.9)	3.7 (6.4)	15.1 (10.8)	< 0.001
**MASCC score, mean (SD)**	20.0 (3.7)	23.3 (1.8)	17.9 (3.0)	< 0.001
≥ 21, N (%)	57 (50)	44 (100)	13 (18.6)	< 0.001
< 21, N (%)	57 (50)	0 (0)	57 (81.4)	
**CISNE score, mean (SD)**	1.9 (1.4)	1.0 (0.6)	2.5 (1.4)	< 0.001
≤ 2, N (%)	80 (70.2)	44 (100)	36 (51.4)	< 0.001
> 2, N (%)	34 (29.8)	0 (0.0)	34 (48.6)	
**Talcott classification group**				< 0.001
4, N (%)	53 (46.5)	44 (100)	9 (12.9)	
1–3, N (%)	61 (53.5)	0 (0)	61 (87.1)	

*SD* standard deviation, *GI* gastrointestinal, *WBC* white blood cell, *ANC* absolute neutrophil count, *CRP* C-reactive protein, *MASCC* Multinational Association of Supportive Care in Cancer, *CISNE* Clinical Index of Stable Febrile Neutropenia.

**Table 2:** Comparisons of clinical features and outcomes between low-risk and high-risk groups.

Variables	Overall (N = 114)	Low-risk (N = 44)	High-risk (N = 70)	*P*
Onset of neutropenia from chemotherapy, day (SD)	9.1 (2.9)	9.4 (2.5)	9.0 (3.1)	0.496
**Hospitalization route, N (%)**				0.001
Outpatient	40 (35.1)	21 (47.7)	19 (27.1)	
ER or already hospitalized	74 (64.9)	23 (52.3)	51 (72.9)	
**Classification of infections, N (%)**				0.003
MDI	13 (11.4)	2 (4.5)	11 (15.7)	0.128
CDI	17 (14.9)	2 (4.5)	15 (21.4)	0.028
Unexplained fever	84 (73.7)	40 (90.9)	44 (62.9)	0.002
**Duration of fever, days (SD)**	2.0 (1.8)	1.5 (1)	2.3 (2.1)	0.014
**Duration of neutropenia, days (SD)**	2.8 (1.6)	2.4 (1)	3.1 (1.9)	0.010
**Duration of hospitalization following febrile neutropenia, day (SD)**	9.6 (10.3)	5.0 (3.4)	12.4 (12.0)	< 0.001
**Initial empirical antibiotic regimen, N (%)**				0.017
TZP monotherapy	63 (55.3)	29 (65.9)	34 (48.6)	0.063
TZP + CIP	19 (16.7)	3 (6.8)	16 (22.9)	0.048
CAZ + isepamicin	11 (9.6)	7 (15.9)	4 (5.7)	0.142
Carbapenem + glycopeptides	6 (5.3)	0 (0)	6 (8.5)	0.118
Others	15 (13.2)	5 (11.4)	10 (14.3)	0.869
**Feasible outpatient management, N (%)**	80 (70.2)	40 (90.9)	40 (57.1)	< 0.001
Successful initial empirical antibiotics, N (%)	88 (77.2)	40 (90.9)	48 (68.6)	0.011
Glycopeptide administration, N (%)	13 (18.6)	0 (0)	13 (11.4)	0.006
Carbapenem administration, N (%)	21 (18.4)	2 (4.5)	19 (27.1)	0.005
Resistant to oral AMC and/or CIP, N (%)	5 (4.4)	0 (0)	5 (4.4)	0.179
Mortality, N (%)	13 (11.4)	0 (0)	13 (18.6)	0.006

*SD* standard deviation, *ER* emergency room, *MDI* microbiologically defined infection, *CDI* clinically defined infection, *TZP* piperacillin/tazobactam, *CIP* ciprofloxacin, *CAZ* ceftazidime, *AMC* amoxicillin/clavulanate.

The patients in the LRG were admitted more often as outpatients (47.7% vs. 19.2%; *P* = 0.001), experienced unexplained fever more frequently (90.9% vs. 62.9%; *P* = 0.002), and had shorter durations of fever (1.5 vs. 2.3; *P* = 0.014), neutropenia (2.4 vs. 3.1 d; *P* = 0.014), and hospitalization after an FN episode (5.0 vs. 12.4 d; *P* < 0.001) than those in the HRG. The LRG patients were more often given TZP monotherapy (65.9% vs. 48.6%; *P* = 0.017) as the initial empirical antibiotic regimen than those in the HRG. Carbapenems plus glycopeptides were administered only in the HRG as an initial empirical antibiotic regimen. The LRG patients demonstrated higher proportions of successful empirical antibiotics treatment (90.9% vs. 68.6%; *P* = 0.011) and feasible outpatient management (90.9% vs. 57.1%; *P* < 0.001) than those in the HRG. All cases of mortality were in the HRG.

### Comparisons between the feasible group and non-feasible group in the HRG

Table 3 shows comparisons of significant variables between the feasible and non-feasible outpatient management group among patients in the HRG. Of the 70 total HRG patients, 40 (57.1%) were classified as the feasible group. There was no significant difference in the mean age and sex ratio between the feasible and non-feasible groups. Patients in the feasible group had a significantly higher white cell count (828.8 vs. 511.0/µL; *P* = 0.003) and lower C-reactive protein (12.3 vs. 18.8 mg/dL; *P* < 0.012) than patients in the non-feasible group. Patients in the feasible group also experienced unexplained fever more frequently (82.5% vs. 36.7%; *P* < 0.001), and they had shorter durations of fever (1.5 vs. 3.2 days; *P* = 0.004) and hospitalization after an FN episode (9.2 vs. 16.6 days; *P* < 0.018) than those in the non-feasible group.

**Table 3:** Comparisons of significant variables between feasible and non-feasible outpatient management group among patients in the high-risk group.

Variables	Feasible (N = 40)	Non-feasible (N = 30)	*P*
**Age (years), mean (SD)**	65.0 (11)	66.5 (11.9)	0.596
**Gender, n (%)**			0.970
Male	28 (70)	20 (66.7)	
Female	12 (30)	10 (33.3)	
**Laboratory results on admission, mean (SD)**			
WBC count (/µL)	828.8 (520.9)	511.0 (328)	0.003
ANC count (/µL)	182.5 (142.2)	169.3 (134.2)	0.696
Hemoglobin (g/dL)	12.7 (21.9)	9.7 (2)	0.392
Platelet count (/µL)	115.5 (92.8)	85.3 (58.1)	0.101
CRP (mg/dL)	12.3 (9.0)	18.8 (12.1)	0.012
**Onset of neutropenia from chemotherapy, day (SD)**	9.7 (2.6)	8.0 (3.4)	0.025
**Hospitalization route, n (%)**			0.100
Outpatient	13 (32.5)	6 (20)	
ER or already hospitalized	27 (67.5)	24 (80)	
**Classification of infections, n (%)**			< 0.001
MDI	2 (5)	9 (30)	0.020
CDI	5 (12.5)	10 (33.3)	0.036
Unexplained fever	33 (82.5)	11 (36.7)	< 0.001
**Duration of fever, days (SD)**	1.5 (1)	3.2 (2.8)	0.004
**Duration of neutropenia, days (SD)**	2.8 (1.6)	3.5 (2.1)	0.086
**Duration of hospitalization following febrile neutropenia, day (SD)**	9.2 (8.4)	16.6 (14.7)	0.018
**Mortality, n (%)**	0 (0)	13 (43.3)	< 0.001

*SD* standard deviation, *WBC* white blood cell, *ANC* absolute neutrophil count, *CRP* C-reactive protein, *MASCC* Multinational Association of Supportive Care in Cancer, *CISNE* Clinical Index of Stable Febrile Neutropenia, *ER* emergency room, *MDI* microbiologically defined infection, *CDI* clinically defined infection.

## Discussion

FN is a potentially life-threatening complication of chemotherapy requiring hospitalization, but only a small number of patients develop serious morbidity^18^. Although the overall mortality of cancer patients with FN is between approximately 3% and 20%, its mortality has diminished steadily because of major advances in the prevention and treatment of FN^19^. However, because of anxiety concerning the safety of patients with FN, even LRG patients with FN have a relatively high hospitalization rate and a long hospital stay^20^. The costs for FN management account for approximately 40%–50% of the total cost of hospitalization for cancer treatment^3,21^. Because of the economic and social burden of FN, it is important to consider the options and determine whether the patient requires hospitalization or not. Thus far, several investigators have developed risk stratification scores to predict the candidate patients for outpatient management of FN. Talcott et al. initially developed a simple risk assessment tool in 1988 based on clinical features present at the onset of FN^22^. Subsequently, the MASCC risk index score and the CISNE score were developed through a multinational collaboration^23,24^. However, all three risk scoring systems have some limitations in predicting successful outcomes for outpatient management^25–28^. To overcome these limitations, in 2018, the ASCO/IDSA updated the clinical practice guideline for outpatient management of FN based on new evidence of risk stratifications for patients who are seemingly stable and at a low risk for FN^1,29^. If this guideline had been implemented in our cancer center, 44 (38.6%) of the hospitalized patients with FN could have been candidates for outpatient management. A total of 80 patients (70.2%), including 40 LRG and 40 HRG patients, were feasible for outpatient management according to our definition regarding treatment outcomes (Table 2). This is a very significant result because many patients who may be treated as outpatients are now hospitalized. Assuming that all other hospitals in Korea are in a similar situation, the significance becomes even greater.

There have not been any studies on the cost-effectiveness of outpatient treatment in patients with FN in Korea, but a study model by Teuffel et al. in Canada suggested that outpatient management is the preferred approach to managing low-risk adult cancer patients in terms of cost-effectiveness^30^. Their Monte Carlo cost–utility model was created to compare the cost of treatment strategies for LRG patients, including inpatient management, early discharge after 48 h of inpatient observation, and outpatient management with oral antibiotics ($13,557 vs. $6115 vs. $3470 [USD])^30^. In a similar retrospective study of cost of LRG patients by Della et al. in the United States, the average cost of care for inpatients was twice that of outpatients ($15,231 vs. $7772 [USD]; *P* < 0.001)^31^. Based on the treatment outcomes of our study, 35.1%–70.2% of inpatients could be considered candidates for outpatient management or early discharge. In conclusion, the classification of LRG patients feasible for outpatient management and transition from inpatient treatment to outpatient treatment can significantly reduce the socioeconomic costs of inpatient care. In Korea, in particular, the application of this guideline is expected to reduce the socioeconomic burden more than in other countries. This is largely because the average length of hospitalization for patients with FN in Korea was 17 days, which was longer than in other countries (e.g., 9.6 days in a study of 91,565 FN patients in the United States)^2,3,32–34^.

In our study, four (9.1%) of the 44 LRG patients were not considered feasible for outpatient management because of modification of antibiotics which could be regarded as treatment failure of initial empirical antibiotics. However, three of these patients recovered from neutropenia within 3 days and were discharged in 4, 5, and 6 days, respectively. They had no cultured microorganisms resistant to amoxicillin/clavulanate or ciprofloxacin, and they could be discharged without complications. Although their conditions did not meet the criteria for outpatient management as we defined, we believe they were LRG candidates for outpatient management or early discharge. A meta-analysis published in 2019 showed that there were no significant differences in treatment failure and mortality between outpatient and inpatient treatment for people with cancer who have low-risk FN^35^. This study, which analyzed ten randomized controlled trials, six in adults (628 participants) and four in children (366 participants), supports our belief.

Forty (57.1%) of the 70 HRG patients were believed to be feasible for outpatient management. The mean length of hospitalization after an FN episode among feasible HRG patients was 9.2 days, which was much shorter than the 16.6 days observed among non-feasible HRG patients (Table 3). In addition, their fever recovered within an average of 1.5 days and neutropenia recovered in an average of 2.8 days, resulting in little difference compared with the LRG. Moreover, while 13 (43.3%) of the 30 patients in the non-feasible group died, none of the 40 patients in the feasible group died (Table 3). These results indicate that some of the HRG patients to whom the updated guideline was applied could be reclassified as patients who could be discharged early, even if it is difficult to apply outpatient management. For example, patients who were considered feasible in the HRG had significantly lower C-reactive protein (CRP) (12.3 vs. 18.8 mg/dL; *P* = 0.012) than the non-feasible HRG patients. Several reports have been published thus far indicating that increased CRP is related to poor prognosis in patients with FN^36–38^. However, to our knowledge, this has never been used in the risk classification criteria of the scoring system or guidelines. Further studies on a more sensitive scoring system using useful criteria, including those currently not proven such as CRP, are needed.

When using MASCC and CISNE risk index scores for applying the updated ASCO/IDSA guidelines to patients with FN, there were difficulties because of subjective or unclear criteria. Regarding the MASCC score, the subjective scoring of “burden of illness” was difficult to use as a criterion because the scores may vary among physicians. In addition, the criteria for previous chronic obstructive pulmonary diseases, cardiovascular disease, and fungal infections related to the MASCC and CISNE scores were sometimes unclear because they could be known only by the patients’ memory or their medical records in other hospitals. Using these subjective or unclear indicators may result in incorrect scoring and misclassification of risks. Particularly, for patients with FN who visit the ER, doctors in the ER cannot know the patients’ past medical history or Eastern Cooperative Oncology Group performance score as well as physicians who usually manage the patient; therefore, it may be difficult to classify the patients into an LRG and to determine their discharge based on the guidelines. Therefore, for patients receiving chemotherapy, it may be helpful to prerecord the medical history required for scoring the MASCC and CISNE on a medical chart. Moreover, it should be noted that subjective judgments by experienced physicians are sometimes much more accurate than objective criteria. However, more research is needed on objective indicators that are easy to use by even less experienced physicians. In addition, if it is not possible to make a decision on outpatient management, multidisciplinary consultation among the departments of oncology, infectious diseases, and emergency medicine will be needed.

### Limitations

There are some limitations in our study. First, because this study is a retrospective cohort study, we were able to assess the risk scores only by an electronic chart review. The clinical judgment criteria, which were suggested by the guideline, were not used in our study because these were difficult to assess retrospectively. To address this limitation, we used three validated risk assessment tools (MASCC score, CISNE score, and Talcott classification) and urine specific gravity as a measurement of hydration status^15,39^. We classified patients as LRG only when the MASCC score, CISNE score, and Talcott classification were compatible with LRG. These criteria were more demanding for LRG than the guidelines, which classified a patient as LRG if either the MASCC score or the Talcott classification was compatible with the CISNE score. Second, this was a single-center study. However, our cancer center is a designated regional cancer center and represents the Daegu-Gyeongsangbuk-do area. According to the internal statistics of the hospital, during the research period of our study (May 1, 2016–April 1, 2018), the hospital treated 37,055 cancer patients aged 18 years or older. Third, we did not investigate the stage of cancer and the strength of anti-cancer drugs. However, type of cancer and other clinical findings such as duration of fever or neutropenia were considered. Fourth, in this study, we arbitrarily defined feasibility for outpatient management of FN based on (1) successful initial empirical antibiotics, (2) no carbapenem or glycopeptide administration, and (3) no isolated microorganism resistant to amoxicillin/clavulanate or ciprofloxacin. The definition of successful initial empirical antibiotics was frequently used in the clinical trial for patients with FN, and we believe the other two criteria were reasonable. Fifth, we did not calculate the sample size. Instead, we determined the observational study period retrospectively from May 1, 2016 to April 1, 2018, because we thought that a longer observational period could make the characteristics of patients and therapeutic strategies too various. Therefore, the results of statistical analysis need to be interpreted cautiously.

## Conclusions

A significant proportion of hospitalized cancer patients treated for fever and neutropenia after chemotherapy could have been feasible candidates for outpatient management. The 2018 ASCO/IDSA guideline can be a useful tool to significantly reduce healthcare labor and the cost for hospitalization. In the future, further prospective studies are needed regarding the application of this guideline for outpatient management of patients with FN.
